# The Importance of a Human Assisted Reproductive Ibero-American Federation: United in Diversity and Strength

**DOI:** 10.5935/1518-0557.20230060

**Published:** 2023

**Authors:** Juan José Espinós

**Affiliations:** 1 President of the Spanish Fertility Society

Latin America and the Iberian Peninsula share an intertwined history that dates back centuries. A legacy of common culture, language, and values unites these lands in a unique richness. Throughout time, they have faced challenges and achieved accomplishments together, and today, more than ever, the need for an Ibero-American Federation focused on Human Reproduction reveals itself as a path towards a promising future.

Unity is strength, and in a globalized world, collaboration and cooperation among nations become essential to address shared challenges, from environmental issues to economic and social crises. An Ibero-American Federation could serve as the platform to strengthen bonds, promote development, and enhance the region’s presence in the international community. By joining forces and sharing knowledge, experiences, and resources, we can achieve results that positively impact the quality of life for many people and couples seeking to conceive a child. Below are some potential implications of this collaboration:

1. Exchange of experiences and best practices: Our countries have different healthcare systems and approaches to assisted reproduction. By collaborating and exchanging experiences, we could learn from each other’s best practices, identify areas for improvement, and develop more effective strategies to address infertility issues and improve treatment outcomes.

2. The Federation as player on the international stage. The union of nations with a shared past and a common vision would allow for greater influence in global issues. Speaking with one voice, the Ibero-American Federation would have a stronger position to advocate for its interests and values in the world arena.

3. Advancements in technology and assisted reproduction techniques: Our collaboration could drive research and development of new technologies and techniques in the field of assisted reproduction. Sharing scientific and medical knowledge would allow specialists to access the latest advancements and apply them more rapidly and effectively for the benefit of patients.

4. Joint research efforts: Combining efforts in scientific research in the field of assisted reproduction would allow addressing complex problems and answering fundamental questions. Collaboration between experts from both regions could lead to significant medical breakthroughs and the development of more effective therapies.

5. Ethical and regulatory framework: Assisted reproduction raises important ethical and legal issues. By collaborating, both regions can share our ethical and regulatory frameworks, seeking to reconcile and improve regulation in this area to ensure the protection of patients’ rights, equitable access to treatments, and preservation of fundamental values.

6. Training and professional development: Collaboration could facilitate the training and professional development of assisted reproduction specialists in both regions. This would ensure greater specialization and knowledge updates, ultimately leading to higher-quality medical care for patients.

Of course, the establishment of an Ibero-American Federation would not be without challenges. Health and social differences could hinder unified decision-making. However, confronting and overcoming these obstacles would be a sign of maturity and the willingness to build a prosperous and united future.

In summary, collaboration between the Iberian Peninsula and Latin America in the field of assisted reproduction has the potential to drive scientific progress, improve access to treatments, strengthen ethical and regulatory frameworks, and provide hope and opportunities to individuals and couples facing infertility issues. Working together in this crucial area of medicine will leverage the strengths and knowledge of both regions for the benefit of global reproductive health.

REDLARA, ALMER, and the Spanish and Portuguese Societies of Fertility have embarked on a journey, to which we hope that the rest of the scientific societies from all the countries in Latin America will soon join, establishing a unique framework to share ideas, projects, and concerns that will elevate the Assisted Human Reproduction of our countries to levels of excellence.

